# Association between a novel nutritional index and coronary heart disease in COPD patients: a retrospective study in China

**DOI:** 10.1186/s12890-026-04124-2

**Published:** 2026-02-24

**Authors:** Lei Yang, Jinxian Yu, Hongmei Yue, DePeng Jiang

**Affiliations:** 1 Department of Respiratory Medicine, The First affiliated Hospital of Lanzhou University, Lanzhou, China; 2 Department of Respiratory Medicine, The Second affiliated Hospital of Chongqing Medical University, Chongqing, China

**Keywords:** Chronic obstructive pulmonary disease, Cardiovascular disease, Triglyceride, Cholesterol, Restricted cubic spline curve

## Abstract

**Background:**

Chronic obstructive pulmonary disease (COPD) commonly coexists with coronary heart disease (CHD). This retrospective study aimed to examine the association between the triglyceride-total cholesterol-body mass index (TCBI) and the CHD in COPD patients.

**Methods:**

This study included 407 participants recruited from the Second Affiliated Hospital of Chongqing Medical University. Logistic regression models were employed to evaluate the association between TCBI and CHD among hospitalized COPD patients undergoing coronary angiography for chest pain. Restricted cubic spline (RCS) analysis was conducted to investigate potential non-linear relationships. Additionally, subgroup analyses were performed to identify variations across different population groups.

**Results:**

The study included 407 participants with a median age of 72 years. After adjusting After adjustment for covariates, each 100-unit increase in TCBI was associated with a significantly lower likelihood of CHD in COPD patients (odds ratio [OR] = 0.927; 95% confidence interval [CI], 0.879–0.976; *P* = 0.004). RCS analysis demonstrated a linear relationship between TCBI and CHD in COPD patients (*P* = 0.021; *P*-nonlinear = 0.986). Subgroup analysis revealed that age ≥ 60 years significantly modified the association between TCBI and CHD in COPD patients (OR = 0.999; 95% CI: 0.998–0.999; *P* < 0.001).

**Conclusion:**

This study suggests a significant association between TCBI and CHD in COPD patients, which may cause selection bias only for this population. Future investigations should validate these findings through well-designed longitudinal studies and multiethnic cohort analyses across diverse populations to enhance the robustness and generalizability of the results.

## Introduction

Chronic obstructive pulmonary disease (COPD) is a complex respiratory disorder characterized by persistent airflow obstruction and chronic pulmonary inflammation [1]. It is frequently accompanied by various comorbidities, with cardiovascular disease (CVD) being particularly prevalent [2, 3]. COPD and CVD share several common risk factors, including advanced age, a history of tobacco smoking or other harmful exposures, and physical inactivity, which contribute to their frequent coexistence in the same patient population [2–4]. Although COPD primarily affects the respiratory system, CVD and malignancies remain leading causes of mortality among affected individuals. Therefore, implementing preventive strategies targeting common CVDs, such as heart failure (HF), coronary heart disease (CHD), hypertension (HTN), atrial fibrillation (AF), and stroke, in COPD patients, or conversely, adopting measures to prevent COPD in individuals with existing CVD, could substantially reduce disability and mortality rates associated with these interrelated conditions [5, 6].

The triglyceride-total cholesterol-body mass index (TCBI) is an emerging nutritional indicator that integrates triglycerides, total cholesterol, and body weight (BW) to provide a comprehensive assessment of metabolic health. Recognized as a significant prognostic marker in CHD [7], TCBI combines lipid profile parameters with body composition metrics to more accurately reflect an individual’s nutritional and metabolic status. Higher TCBI values have been associated with improved metabolic outcomes, a reduced risk of cognitive decline, and a lower incidence of stroke and stroke-related pneumonia [8–10]. Furthermore, TCBI serves as a valuable predictor of prognosis in patients with HF and in those receiving mechanical circulatory support during critical illness. Additionally, the individual components of TCBI have been linked to depressive symptoms, which themselves are associated with frailty and nutritional deficiencies [11–13]. Malnutrition has been associated with unfavorable clinical outcomes in chronic obstructive pulmonary disease, such as deteriorated lung function, elevated exacerbation frequency, extended hospital stays, and increased mortality rates [14, 15]. It exerts a significant influence on muscle energetics, exercise tolerance, and the severity of dyspnea, which are three aspects vital to the overall health and quality of life of COPD patients [16]. TCBI, as a nutritional indicator, has been increasingly recognized for its potential utility in assessing the nutritional and overall health status of patients with COPD [17]. By integrating multiple physiological parameters, TCBI provides a comprehensive assessment of an individual’s nutritional condition and facilitates a better understanding of the complex interactions among malnutrition, inflammation, oxidative stress, and immune dysfunction in COPD.

However, the relationship between TCBI and CHD in COPD patients remains unclear. Therefore, this retrospective cohort study aimed to investigate the potential association between TCBI and CHD in COPD patients, which may cause selection bias only for this population.

## Materials and methods

### Research participants

This cross-sectional study enrolled 407 patients diagnosed with COPD based on pulmonary function testing. All participants underwent coronary angiography for chest pain at the Second Affiliated Hospital of Chongqing Medical University between January 2020 and December 2024. Based on angiographic findings, patients were classified into two groups: COPD with CHD (COPD + CHD) and COPD only. COPD diagnosis was confirmed by pulmonary function testing, characterized by clinical symptoms such as dyspnea or wheezing and a post-bronchodilator forced expiratory volume in one second to forced vital capacity ratio (FEV_1_/FVC) below 0.7. Participants with a history of malignancy, autoimmune disorders, acute or chronic infections, or other pulmonary diseases were excluded. The study protocol was approved by the Ethics Committee of the Second Affiliated Hospital of Chongqing Medical University and conducted in accordance with institutional ethical guidelines.

### Calculation of TCBI

The TCBI was computed using the formula defined as $TCBI=\frac{TG\:(mg/dL)\times\:{TC}(mg/dL)\times {BW}(kg)}{1,000}$ [18].

### Statistical analysis

Statistical analyses were performed using R version 4.2.2 and SPSS version 26.0. Descriptive statistics are presented as adjusted proportions (%) and weighted means with corresponding measures of dispersion. Categorical variables were compared using the chi-square test, while continuous variables were analyzed using the Wilcoxon test. Three regression models were developed: Model I (unadjusted), Model II (adjusted for age and sex), and Model III (adjusted for age, sex, smoking history, drinking history, hypertension, diabetes, platelet count, systolic and diastolic blood pressure, height, neutrophil count, and lymphocyte count). Restricted cubic spline (RCS) models were employed to evaluate the relationship between TCBI and CHD among patients with COPD. In addition, subgroup analyses were performed to examine potential variations in this association across different population subgroups. A p-value of less than 0.05 was considered statistically significant.

## Results

### Participant baseline characteristics

The median age of all participants was 72 years in this study. Males accounted for 79.36% of the study population, while 64.37% were smokers and 38.82% were drinkers. The median TCBI value was 509.02. Statistically significant differences (*P* < 0.05) were observed in several variables, including sex, smoking history, diastolic blood pressure, neutrophil count, monocyte count, and high-density lipoprotein cholesterol levels, as detailed in Table 1.

**Table 1:** Baseline characteristics of all participants

Variable	Overall (*N* = 407)	COPD + CHD (*N* = 248)	COPD (*N* = 159 )	*P* value
^*^Age(years)	72.00 (67.00–77.00)	72.00 (67.75–77.00)	72.00 (67.00–76.00)	0.090
Gender(%)				< 0.001
	84 (20.64%)	36 (14.52%)	48 (30.19%)	
	323 (79.36%)	212(85.48%)	111(69.81%)	
Hypertension(%)				0.170
	189 (46.44%)	108 (43.55%)	81(50.94%)	
	218 (53.56%)	140 (56.45%)	78 (49.06%)	
Diabetes(%)				1.000
	326(80.10%)	199 (80.24%)	127(79.87%)	
	81(19.90%)	49 (19.76%)	32(20.13%)	
Smoking(%)				< 0.001
	145(35.63%)	73(29.44%)	72(45.28%)	
	262(64.37%)	175(70.56%)	87(54.72%)	
Drinking(%)				0.380
	249(61.18%)	147(59.27%)	102(64.15%)	
	158(38.82%)	101(40.73%)	57(35.85%)	
^#^Systolic pressure(mmHg)	133.82 ± 20.42	133.68 ± 20.86	134.04 ± 19.79	0.860
Diastolic pressure(mmHg)	80.00 (73.00–88.00)	78.00 (72.00–86.25)	83.00 (74.00–88.50)	0.010
^#^Weight(kg)	63.60 ± 11.35	63.40 ± 11.29	63.92 ± 11.48	0.650
^*^Height(cm)	165.00 (159.00–170.00)	165.00 (160.00–170.00)	165.00 (158.00–169.00)	0.200
^*^Platelets(10*9/L)	188.00 (154.50–224.50)	187.00 (153.75–220.50)	192.00 (157.50–231.50)	0.280
^*^Neutrophil(10*9/L)	4.29 (3.50–5.72)	4.56 (3.62–6.15)	4.00 (3.33–5.20)	< 0.001
^*^Lymphocytes(10*9/L)	1.43 (1.05–1.80)	1.42 (1.00–1.75)	1.44 (1.10–1.86)	0.090
^*^Monocyte(10*9/L)	0.490 (0.370–0.610)	0.510 (0.400–0.630)	0.450 (0.330–0.590)	0.002
^*^Total cholesterol(mmol/L)	4.47 (3.70–5.17)	4.47 (3.68–5.29)	4.46 (3.80–5.06)	0.750
^*^Triglycerides(mmol/L)	1.18 (0.90–1.73)	1.22 (0.96–1.82)	1.17 (0.84–1.58)	0.070
^*^High-density lipoprotein cholesterol(mmol/L)	1.21 (1.04–1.40)	1.19 (1.01–1.38)	1.25 (1.07–1.43)	0.020
^*^Low-density lipoprotein cholesterol(mmol/L)	2.51 (1.94–3.04)	2.53 (1.93–3.06)	2.48 (1.94–2.96)	0.580
^*^TCBI	509.02 (332.71–793.16)	536.39 (341.94–843.06)	474.32 (327.10–759.84)	0.140

For categorical variables, Fisher’s Exact Test and the Chi-Square Test of Independence were employed, whereas for continuous variables, the Mann - Whitney U Test (^*^Median [IQR]) and the T - Test (^#^Mean ± SD) were utilized

*TCBI* Triglyceride-total cholesterol-body mass index, *COPD* Chronic obstructive pulmonary disease, *CHD* coronary heart disease

### Analysis of the association between TCBI and COPD patients with CHD

After adjusting for relevant covariates, each 100-unit increase in TCBI was associated with a significantly lower likelihood of CHD among patients with COPD (OR = 0.927; 95% CI, 0.879–0.976; *P* = 0.004), as detailed in Table 2. Moreover, individuals in the highest quartile (Quartile 4) had a 49.5% lower odds of developing CHD compared to those in the lowest quartile (Quartile 1) (OR = 0.505; 95% CI: 0.258–0.989; *P* = 0.046).

**Table 2:** Multivariable logistic regression models examining the association between TCBI and the CHD in COPD patients

Variable	Model1		Model2		Model3	
	OR (95% CI)	*P*	OR (95% CI)	*P*	OR (95% CI)	*P*
TCBI(each-100unit)	0.951 (0.909, 0.996)	0.031	0.933 (0.889,0.980)	0.006	0.927 (0.879,0.976)	0.004
TCBI (Q1)	Reference		Reference		Reference	
TCBI (Q2)	1.271 (0.730, 2.215)	0.397	1.282 (0.724,2.268)	0.395	1.148 (0.629,2.093)	0.653
TCBI (Q3)	0.789 (0.446, 1.395)	0.415	0.718 (0.398,1.294)	0.270	0.612 (0.325,1.152)	0.128
TCBI (Q4)	0.812 (0.460, 1.431)	0.470	0.644 (0.356,1.165)	0.146	0.505 (0.258,0.989)	0.046
p for trend		0.228		0.048		0.015

Model I featured no adjustment for covariates, whereas Model II incorporated adjustments for age and sex. Moving on to Model III, a comprehensive adjustment was made for age, sex, smoking history, drinking history, hypertension, diabetes, platelets, systolic pressure, diastolic pressure, height, neutrophil, lymphocytes

*TCBI* Triglyceride - total cholesterol-body mass index, *OR* Odds ratio, *CI* Confidence intervals

### Analysis of RCS curve

**Fig. 1:**
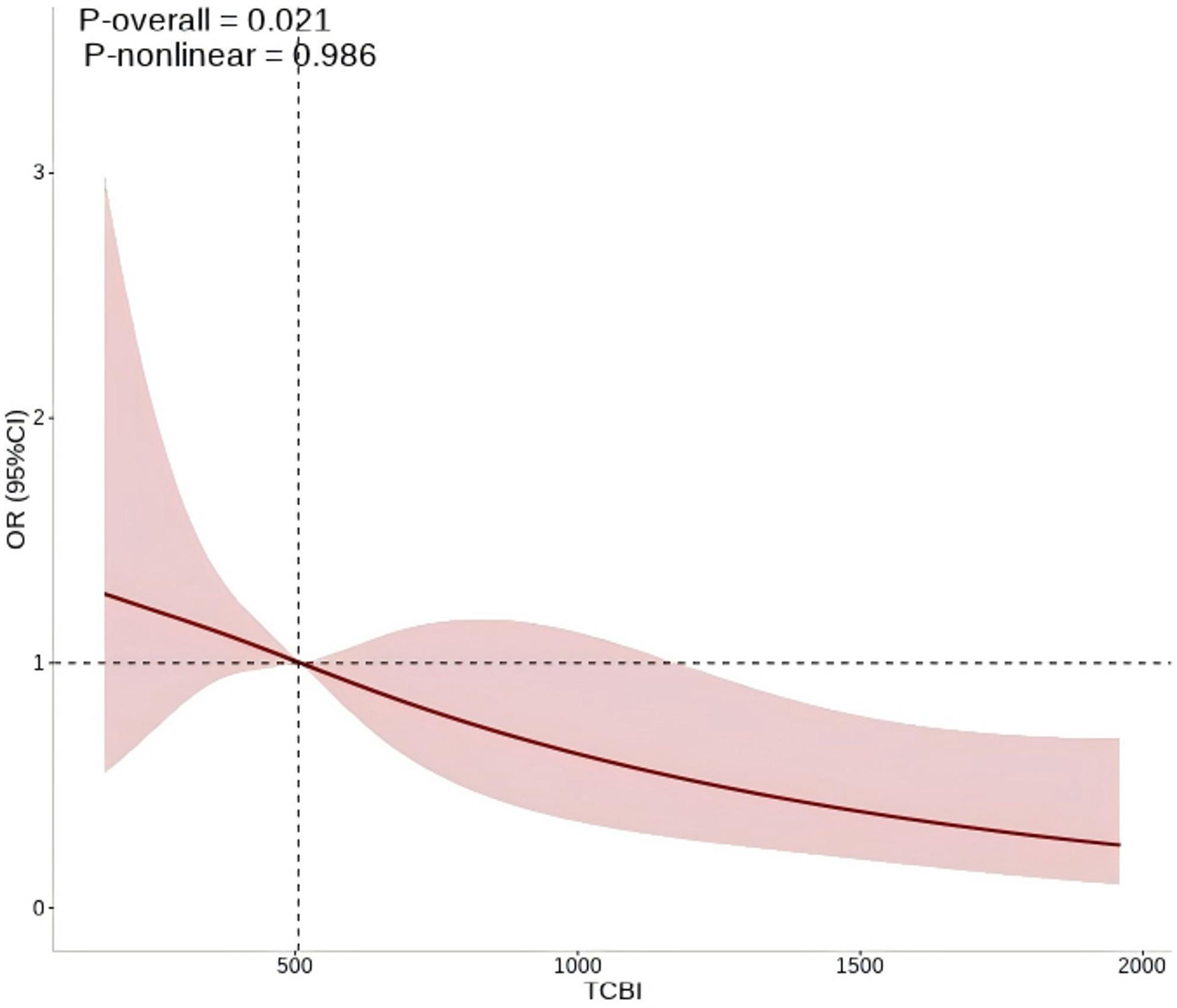
Adjusted RCS curve depicting the linear association between TCBI (per 100-unit increase) and the odds of CHD in COPD patiens. The pink bars show the fitted 95% confidence intervals (95% CI) and the restricted cubic spline are shown in red. TCBI, triglyceride-total cholesterol-body mass index; CHD, coronary heart disease; COPD, chronic obstructive pulmonary disease

The RCS analysis did not detect evidence of a non-linear association between TCBI and CHD risk (P for nonlinearity = 0.986), supporting the use of a linear model, as shown Fig. 1.

### Subgroup analysis

To comprehensively evaluate the robustness of the association between TCBI and CHD in COPD patients and to explore potential subgroup differences, multi-subgroup analyses with interaction tests were conducted, adjusting for multiple covariates (Table 3). A consistent and statistically significant association was observed across most subgroups. However, age significantly modified this relationship (interaction *P* = 0.050). Specifically, the association between TCBI and CHD in COPD patients was among patients aged 60 years and older (OR = 0.999; 95% CI: 0.998–0.999; *P* < 0.001). However, the confidence intervals for the younger group are very wide due to small sample size, the association is uncertain.

**Table 3:** Subgroup analysis of TCBI - COPD patients with CHD correlation

Various	OR(95%CI) *P* value	*P* for interaction
Age group(%)		
< 60 years	**1.004 (1.000-1.008) 0.079**	**0.050**
≥ 60 years	**0.999 (0.998–0.999) < 0.001**	
Gender(%)		0.946
Male	0.999 (0.997-1.000) 0.032	
Female	0.999 (0.999-1.000) 0.023	
BMI group(%)		
BMI ≤ 18.5 kg/m^2^	1.055 (0.952–1.169) 0.305	0.315
18.5 < BMI ≤ 25.5 kg/m^2^	0.999 (0.998-1.000) 0.049	
BMI ≥ 25.5 kg/m^2^	0.999 (0.998-1.000) 0.041	
Smoking(%)		0.835
No	0.999 (0.998-1.000) 0.012	
Yes	0.999 (0.999-1.000) 0.086	
Drinking(%)		0.472
No	0.999 (0.998-1.000) 0.005	
Yes	0.999 (0.999-1.000) 0.199	
Hypertension(%)		0.493
No	0.999 (0.998-1.000) 0.019	
Yes	0.999 (0.998-1.000) 0.053	
Diabetes(%)		0.149
No	0.999 (0.998-1.000) 0.004	
Yes	1.000 (0.999–1.001) 0.394	

*TCBI* Triglyceride-total cholesterol-body mass index, *CHD* Coronary heart disease, *COPD* Chronic obstructive pulmonary disease, *OR* Odds ratio, *CI* Confidence intervals

## Discussion

To our knowledge, this study is the first to investigate the association between TCBI and CHD in COPD patients using a retrospective cohort design, we found that each 100-unit increase in TCBI was associated with a significantly lower likelihood of CHD among patients with COPD (OR = 0.927; 95% CI, 0.879–0.976; *P* = 0.004). Subgroup analysis further demonstrated that this association is significantly modified by patient age, with a stronger relationship observed in older individuals.

In recent years, increasing attention has been focused on the relationship between nutritional status and the occurrence and prognosis of various diseases. Traditional nutritional assessment tools, such as the Prognostic Nutritional Index (PNI), Controlling Nutritional Status (COUNT), and Geriatric Nutritional Risk Index (GNRI), have seen limited clinical use due to their complexity and reliance on multiple parameters [19–21]. In contrast, the TCBI is a recently introduced, simplified nutritional indicator that integrates serum triglycerides (TG), total cholesterol (TC), and BW [7]. As a composite measure, TCBI effectively reflects both nutritional status and metabolic reserves, making it a valuable metric for assessing health risks related to malnutrition and metabolic imbalance [12, 22]. Compared to traditional indices like PNI, COUNT, and GNRI, TCBI offers greater practicality and ease of use by relying on routinely measured laboratory variables, enhancing its clinical applicability [23, 24]. Emerging evidence links TCBI to a variety of health outcomes. Liu et al. reported an inverse association between TCBI and cognitive impairment among middle-aged and older Chinese adults, particularly in those aged ≥ 60 years, non-drinkers, and socially active individuals [8]. Another study found that each unit increase in TCBI corresponds to a 28% reduction in the risk of sarcopenia [25]. Furthermore, TCBI has been independently negatively correlated with stroke prevalence, with cumulative TCBI showing a non-linear relationship with stroke risk, especially among hypertensive individuals younger than 60 years [9, 26]. Additionally, TCBI has been associated with both short- and long-term adverse outcomes in patients with acute ischemic stroke (AIS) [27]. Previous studies have demonstrated that malnutrition is closely associated with the progression of COPD [28]. However, inflammatory processes, oxidative stress, and immune dysfunction should not be overlooked [29, 30]. Nutritional status is modulated through inflammatory and oxidative stress pathways, which are closely linked to immune system regulation [31]. For example, malnourished patients with COPD experience heightened levels of inflammation and oxidative stress [15], and weight loss in this population may be, at least in part, attributable to inflammatory processes. A previous study demonstrated that multiple nutritional indicators are associated with COPD and mortality [32]. These findings suggest that interventions targeting metabolic dysregulation and hypertension control may help mitigate stroke risk in aging populations. However, the relationship between TCBI and CHD in COPD patients remains unclear. To the best of our knowledge, this study is the first to investigate the association between TCBI and CHD in COPD patients.

Previous research has shown that lower TCBI values are associated with poorer survival outcomes, including increased all-cause, cardiovascular, and cancer-related mortality. Similar trends have been observed in patients undergoing percutaneous coronary intervention (PCI) [33, 34]. However, multivariate analyses have demonstrated that TCBI is significantly linked only to reduced all-cause mortality, with no meaningful association with cardiovascular or cancer-specific mortality risks. Conversely, some studies have reported a significant positive correlation between TCBI and CVD mortality, while finding no association between TCBI and the atherosclerosis index, an important marker of CVD risk [35]. Additionally, prior investigations have not identified any significant relationship between TCBI and disease occurrence or mortality in COPD patients [17]. In contrast, our study reveals that higher TCBI levels are associated with lower odds of CHD in COPD patients. This study focused on symptomatic hospitalized patients undergoing angiography, a population that differs substantially from the community population, post-PCI patients, and the general elderly population examined in previous studies. From the perspective of disease severity, patients in the present study likely had more advanced conditions, with overt cardiovascular symptoms necessitating angiography for diagnosis. In contrast, community populations are typically in earlier or asymptomatic stages of disease, post-PCI patients are generally in a recovery phase, and the general elderly population may only exhibit potential cardiovascular risk factors without meeting the clinical threshold for angiography. These differences may result in divergent associations between TCBI and CHD across studies, reflecting fundamentally distinct association patterns between severe and mild disease groups. In addition, patients in this study underwent angiography and hospitalization due to clinical symptoms, introducing potential selection bias, as only individuals with prominent symptoms or those deemed by physicians to require angiography were included. By comparison, community-based populations in previous studies were typically selected through random sampling, leading to substantially different inclusion criteria. Such selection bias may affect the representativeness of the study populations and, consequently, influence the observed association between TCBI and CHD, contributing to inconsistent findings across studies. Moreover, patients in the present study may have already received various treatments prior to enrollment, which could modify disease progression and TCBI levels. In contrast, populations in previous studies differed in both treatment modalities and treatment timing. For example, patients undergoing specific interventional therapies after PCI often exhibit marked differences in physical condition and biomarker profiles compared with the population examined in this study. These treatment-related differences may confound the assessment of the true association between TCBI and CHD, thereby leading to divergent conclusions across studies.

Previous studies have suggested that body mass index (BMI) plays a mediating role between COPD and CVDs [36]. Therefore, TCBI may influence the development of cardiovascular complications in COPD by affecting obesity-related metabolic dysregulation. Future research should further investigate the underlying molecular mechanisms, such as systemic inflammation, lipid metabolism disturbances, and oxidative stress, through which TCBI modulates metabolic homeostasis and, in turn, impacts cardiovascular risk. Additionally, since statins and certain pulmonary therapies can simultaneously affect metabolic pathways and cardiovascular outcomes [37, 38], future large-scale longitudinal studies should incorporate treatment-related variables to evaluate their interaction with TCBI.

This study identified age as a key factor influencing the relationship between TCBI and CHD in COPD patients, with the association particularly pronounced in individuals aged over 60 years. Similarly, previous research has demonstrated a significant negative correlation between TCBI and cognitive impairment in populations aged ≥ 60 years, suggesting that the impact of TCBI on health outcomes may intensify with advancing age [8]. This pattern may be attributed to age-related declines in metabolic efficiency and the cumulative effects of chronic inflammation, which together increase cardiovascular vulnerability. In elderly individuals, the stronger association between TCBI and CHD events in COPD patients highlights the importance of enhanced metabolic monitoring and timely preventive interventions in this demographic.

This study has several limitations. First, its cross-sectional design limits the ability to infer causality and may reduce the generalizability of the findings. Second, this study exclusively included hospitalized patients with COPD who underwent coronary angiography for chest pain, which may result in the conclusion being applicable only to this population group and limit the generalizability of the findings to the broader COPD population, particularly asymptomatic individuals or those with atypical presentations. Third, several key variables, including COPD severity indicators (e.g., GOLD stage, forced expiratory volume in 1 s [FEV_1_] percentage, and history of acute exacerbations) and medication use (e.g., statins, antiplatelet agents, beta-blockers, and inhaled corticosteroids or bronchodilators), were not available for analysis, both directly influence cardiovascular risk, nutritional status, and lipid profiles (TCBI components), potentially resulting in residual confounding that may affect the accuracy of the results. In addition, the study relied solely on cell count data derived from complete blood counts, while important inflammatory biomarkers, such as C-reactive protein, which may have led to an incomplete assessment of inflammation severity, potentially compromising the precision of disease severity and risk evaluation, as well as the overall accuracy of the findings. Finally, potential inaccuracies during data collection could have affected the reliability of the results. Future research should address these limitations by employing multicenter, prospective study designs to enhance external validity and by including more ethnically diverse populations. Such studies should clearly specify key data to be collected and reported, including detailed lung function measures, comprehensive medication histories, and inflammatory biomarkers, as well as define the target populations, such as patients with varying degrees of COPD severity and community-dwelling individuals, in order to minimize selection bias.

In summary, this study suggests a significant association between TCBI and CHD in COPD patients, which may cause selection bias only for this population. The analyses were exploratory in nature, and the findings should be interpreted as hypothesis-generating rather than confirmatory. Future studies should seek to validate these results through well-designed longitudinal investigations and multiethnic cohort analyses across diverse populations to enhance the robustness and generalizability of the conclusions.

## Data Availability

The data that support the findings of this study are available on request from the corresponding author. The data are not publicly available due to privacy or ethical restrictions.
